# Evolution of Hospitalisation Due to Stroke in Italy Before and After the Outbreak of the COVID-19 Epidemic: A Population-Based Study Using Administrative Data

**DOI:** 10.3390/jcm14020353

**Published:** 2025-01-08

**Authors:** Emanuele Amodio, Gabriele Di Maria, Manuela Lodico, Dario Genovese, Vito M. R. Muggeo, Laura Maniscalco, Michela Conti, Maria Sergio, Antonio Cascio, Antonino Tuttolomondo, Domenica Matranga, Francesco Vitale, Marco Enea

**Affiliations:** 1 Department of Health Promotion, Mother and Child Care, Internal Medicine and Medical Specialties, University of Palermo, 90127 Palermo, Italy; emanuele.amodio@unipa.it (E.A.); gabriele.dimaria@unipa.it (G.D.M.); manuela.lodico@you.unipa.it (M.L.); dario.genovese@unipa.it (D.G.); laura.maniscalco04@unipa.it (L.M.); maria.sergio@unipa.it (M.S.); antonio.cascio03@unipa.it (A.C.); antonino.tuttolomondo@unipa.it (A.T.); domenica.matranga@unipa.it (D.M.); francesco.vitale@unipa.it (F.V.); 2 Department of Economics, Business and Statistics, University of Palermo, 90128 Palermo, Italy; vito.muggeo@unipa.it; 3 Azienda Ospedaliera Ospedali Riuniti (AOOR) Villa Sofia Cervello, 90146 Palermo, Italy; michelaconti@virgilio.it

**Keywords:** ischaemic stroke, stroke hospitalisation incidence, COVID-19, sex and age differences, retrospective cohort study, registry data

## Abstract

**Background/Objectives**: Stroke is a leading cause of mortality and disability worldwide, ranking as the second most common cause of death and the third in disability-adjusted life-years lost. Ischaemic stroke, which constitutes the majority of cases, poses significant public health and economic challenges. This study evaluates trends in ischaemic stroke hospitalisations in Italy from 2008 to 2022, focusing on differences before and after the COVID-19 pandemic. **Methods**: We analysed ischaemic stroke hospitalisations among individuals admitted through emergency services using Italian hospital discharge records from 2008 to 2022. Poisson Inverse Gaussian regression was employed to assess hospitalisation trends, accounting for age, sex, and geographic variations. **Results**: Among 1,689,844 ischaemic stroke hospitalisations, there was a marked age-related increase, particularly among individuals aged 74 and older, with males consistently showing higher rates. Hospitalisation trends demonstrated a 20% reduction over 15 years, suggesting improvements in stroke prevention and treatment. However, there was a slight increase in rates during the COVID-19 period, despite the overall declining trend, highlighting the potential healthcare challenges experienced during the pandemic. Multivariable analysis confirmed age and male sex as significant risk factors. **Conclusions**: This study underscores the age-related increase in stroke hospitalisation rates, emphasising the need for targeted prevention strategies for elderly populations. The overall reduction in stroke hospitalisation rates reflects advancements made in healthcare, although the impact of COVID-19 on access to stroke care is evident. Future policies must address the pandemic’s effects on stroke care continuity and prioritise interventions tailored to age and sex.

## 1. Introduction

A stroke is a cerebrovascular episode that occurs when the supply of oxygenated blood to the brain is interrupted. It may occur due to the abrupt occlusion of a blood vessel by a thrombus (ischaemic stroke), or the rupture of a blood vessel supplying the brain (haemorrhagic stroke) [1]. Ischaemic strokes constitute 80% of all stroke occurrences, whilst haemorrhaging strokes represent the remaining 20%. Typically, this event causes irreversible damage to brain tissue due to an interruption of blood supply, frequently culminating in necrosis.

Stroke symptoms emerge abruptly, and might include muscular weakness, paralysis, speech impairments, confusion, and visual disturbances. Timely identification and immediate medical intervention are essential to reduce cerebral damage and its repercussions.

Stroke remains the second leading cause of mortality, and the third principal cause of combined mortality and disability (measured in disability-adjusted life-years) worldwide [2]. The estimated global costs associated with stroke exceed USD 721 billion [2]. Between 1990 and 2019, the burden of stroke has markedly escalated, with a 70.0% increase in cases and a 43.0% rise in stroke-related deaths [2].

In 2017, the total economic burden of stroke across 32 European nations was 60 billion euros, with healthcare costs being EUR 27 billion. The majority of healthcare expenses arose from inpatient care, followed by outpatient and primary care. Informal caregiving amounted to EUR 16 billion, signifying considerable dependency on familial support. The economic repercussions were exacerbated by EUR 6.3 billion in productivity losses attributable to stroke-related morbidity and mortality, highlighting the substantial direct and indirect costs associated with stroke [3].

As populations expand and individuals age, the incidence of stroke occurrences and their long-term consequences, along with associated costs, are anticipated to rise considerably [4].

In 2021, 10–12% of all annual deaths worldwide were attributable to stroke [2]. The stroke mortality rate was 20–30% within 30 days post-event and 40–50% after one year, with 75% of individuals who survived experiencing some degree of disability, half of which resulted in a loss of independence [5]. This medical condition is one of the most prevalent and frequent, having a clear correlation with age. The incidence notably rises from the age of 55, and after 65 years, the increase becomes exponential [1]. Other principal modifiable risk factors include hypertension, diabetes mellitus, use of tobacco products, and hyperlipidaemia, alongside lifestyle-related factors such as obesity, an inadequate diet, and physical inactivity [6].

Furthermore, SARS-CoV-2 infection has been identified as a significant risk factor for stroke [7]. Multiple mechanisms associated with immunomodulated thrombosis, alterations in coagulation pathways, the renin-angiotensin system, and the influence of the virus on cardiac and cerebral tissues may contribute to the pathophysiology of ischaemic stroke in COVID-19 patients [8]. Indeed, SARS-CoV-2 virus contributes to ischaemic stroke due to the spike protein’s strong affinity for the human ACE2 receptor, resulting in endothelial dysfunction, cellular apoptosis, and neuronal damage, as well as facilitating excessive thrombin production and inhibiting fibrinolysis [9]. In COVID-19 patients, the hypercoagulable state is exacerbated by hypoxaemia, which is prevalent in severe cases, which elevates blood viscosity and activates hypoxia-related genes, influencing coagulation and fibrinolysis processes, hence increasing the risk of thrombotic events [10,11].

Multicentre studies and meta-analyses indicate that the incidence of stroke in individuals with COVID-19 ranges from 0.5% to 4.5%, with the risk of acute stroke escalating in accordance with the severity of the condition [7,12]. A population-based study indicated that the COVID-19 pandemic may have indirectly affected stroke care procedures, leading to delays in identification and treatment, as well as a reduction in hospital admissions for stroke, particularly during the first waves of the pandemic [13]. These findings underscore the necessity to investigate the impact of the COVID-19 pandemic on stroke hospitalisation rates in Italy, and its possible implications for long-term public health effects.

This observational study aimed to evaluate the trend of ischaemic stroke hospitalisations in Italy over a 15-year period, with particular emphasis on the pre- and post-pandemic periods, considering the overall impact on the general population, the economic burden, and the commitment of healthcare personnel associated with acute ischaemic stroke, including acute hospitalisation and long-term rehabilitation.

## 2. Materials and Methods

This retrospective cohort study examines data on stroke hospitalisations obtained from the Italian hospital discharge records (HDRs) database, which collects detailed information on patients admitted to all hospitals across Italy.

### 2.1. Study Population and Period

For the purposes of this study, we evaluated all hospitalisations that occurred in Italy between 1 January 2008 and 31 December 2022, covering an average at-risk population of approximately 60,000,000 individuals during the study period.

### 2.2. Data Sources and Variables

The HDRs database provided comprehensive data for each hospitalisation, including:

- Demographic information: sex, date of birth, and region of residency.
- Hospitalisation details: admission and discharge dates, discharge status (categorised as “discharged/transferred” or “expired”).
- Clinical information: up to six discharge diagnoses, including one principal diagnosis, and up to five secondary diagnoses, all coded according to the International Classification of Diseases, Ninth Revision, Clinical Modification (ICD-9-CM).

Cases of ischaemic stroke were identified based on the presence of ICD-9-CM codes 433.x-434.x, appearing in either the principal or secondary diagnosis fields.

### 2.3. Inclusion and Exclusion Criteria

To ensure consistency and focus on acute cases, we applied the following criteria:

- Only emergency admissions were included in the analysis.
- Elective hospitalisations were excluded, as these cases typically involve pre-planned procedures or non-urgent care.

### 2.4. Population and Regional Data

Population statistics, including age- and sex-specific data, were sourced from the demo.istat web section of the Italian National Institute of Statistics (ISTAT). These data were used to calculate hospitalisation rates and adjust for demographic trends over the study period.

### 2.5. Ethical Approval

This study was approved by the Ethics Committee of the A.O.U.P. University Hospital of Palermo (approval code: CEP/2021/08; approval date: 15 September 2021).

### 2.6. Statistical Analysis

Hospitalisations were summarised as absolute cases and rates (cases × 10,000 at risk people per year).

Hospitalisation rates per 10,000 people were determined from the demo.istat census population [14]. To be consistent with the demo.istat census population, patients were divided by sex, age groups of 0–14, 15–24, 25–44, 45–64, 65–74, and >74, and region of residence. Moreover, to capture seasonality in the temporal trends, hospitalisations were also grouped by season. For each category of sex, age group, and season, the stroke hospitalisation rate temporal trends were graphically examined.

Given that the risk estimate could be significantly influenced by spatial (regional) and temporal factors, as well as by the heterogeneity of the population in each Italian region, a Poisson-inverse Gaussian (PIG) regression random-effects model was employed, where the region was included as a random intercept and the national population was used as an offset. The model’s linear predictor was developed by estimating the change and jump points of the log incidence with time (year). A linear predictor for the PIG’s scale parameter was also specified to improve the goodness-of-fit of the entire model. The validity of this approach has been confirmed via diagnostic examination of the model residuals. Results describing temporal trends were presented as relative risk (RR), annual percentage change (APC), and average annual percentage change (AAPC), along with 95% confidence intervals (C.I.). Additional information regarding the model specification is provided in the Supplementary Materials. A *p*-value < 0.05 was considered statistically significant.

Analyses were performed using R Software (version 4.0.5; R Foundation for Statistical Computing (Vienna, Austria)), together with the packages “GAMLSS” [15] and “segmented” [16].

## 3. Results

Table 1 indicates that, throughout the study period, there were 1,689,844 hospitalisations for stroke in Italy, resulting in a rate of 18.76 cases per 10,000 person-years. Hospitalisation rates were similar throughout seasons, with slightly higher rates in winter (19.10 per 10,000 person-years) and spring (19.06 per 10,000 person-years), compared to summer (18.11 per 10,000 person-years) and autumn (18.77 per 10,000 person-years). The incidence rate for males was 19.96 per 10,000 person-years, beyond the rate for females at 17.63 per 10,000 person-years, with a significant increase in hospitalisation rates observed with advancing age. The rate was greatest among individuals over 74 years (105.97 per 10,000 person-years), whereas it was significantly lower in younger age groups (0.10 per 10,000 person-years for ages 0–14).

Hospitalisation rates exhibited minor fluctuations from 2008 to 2022, showing an overall decline, with rates ranging from 15.68 to 16.12 per 10,000 person-years between 2020 and 2022.

**Figure 1:**
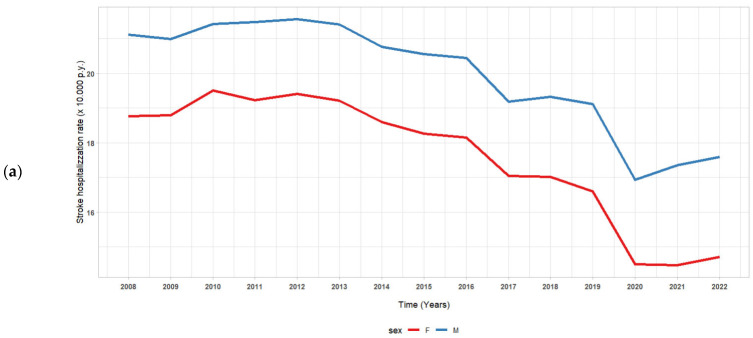

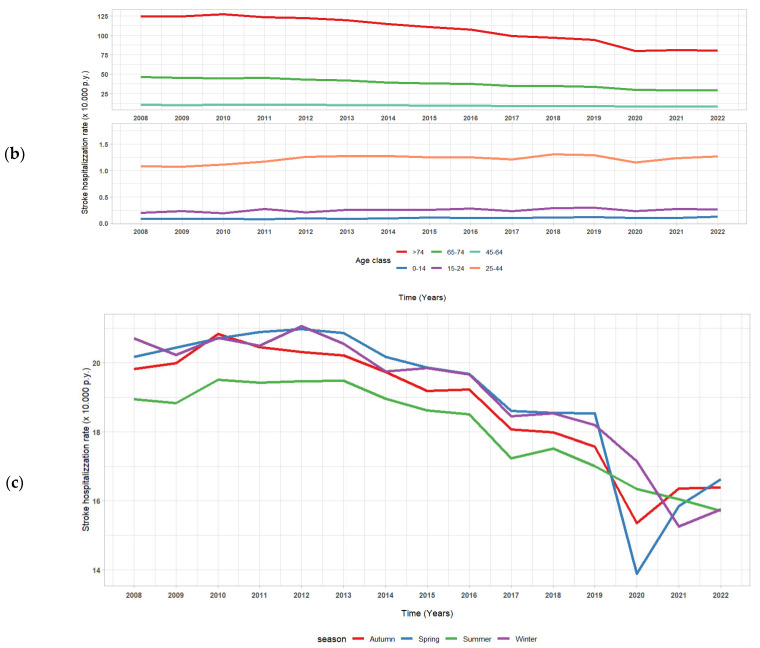
(**a**) Trends of hospitalisations due to stroke stratified by sex; (**b**) trends of hospitalisations due to stroke stratified by age; (**c**) trends of hospitalisations due to stroke stratified by seasons.

Figure 1a depicts the trend in stroke hospitalisation rates (per 10,000 person-years) from 2008 to 2022, stratified by sex, with males represented in blue and females in red. There has been a consistent decrease in stroke hospitalisation rates for both sexes over the years. Figure 1b illustrates stroke hospitalisation rates from 2008 to 2022, categorised by age group.

Individuals aged 74 or older (red line) consistently exhibit the highest rates of stroke hospitalisation, peaking at over 120 in 2008 and gradually decreasing over the years, until stabilising at approximately 85 per 10,000 person-years by 2022. Individuals aged 65–74 years (green line) show elevated hospitalisation rates, exhibiting a declining trend from approximately 40 per 10,000 person-years in 2008 to just above 30 in 2022. The age group 45–64 (orange line) exhibits a consistently stable rate, approximately 10 per 10,000 person-years, with a minor drop noted with time.

**Figure 2:**
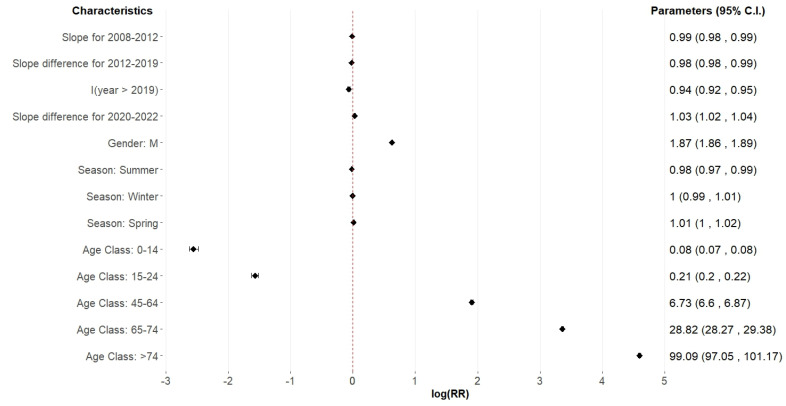
Forest plot of the multivariable model on factors involved in changes in hospitalisation rates for stroke over time. The reference categories for the RRs are sex = F, season = spring, and age class = 25–44 years.

The results of the multivariable model for factors influencing changes in stroke hospitalisation rates over time are illustrated in the forest plot in Figure 2, and are also reported in log-scale, along with standard errors and *p*-values, in Table 2. The temporal analysis revealed two notable shifts: a change point in 2012, and a simultaneous jump and change point in 2019, which modified the slope of the linear influence of the year variable. From 2008 to 2012, there was an annual decrease in the risk of hospitalisation due to stroke (RR = 0.99, 95% CI 0.99–0.99, *p*-value < 0.001). The downward linear trend accelerated from 2013 to 2019, with a multiplicative factor of RR = 0.98 (95% CI 0.98–0.99, *p*-value < 0.001). In 2019, the linear trend was interrupted by a notable decline in hospitalisation rates (RR = 0.91, 95% CI = 0.89–0.93, *p*-value < 0.001). The sequence of reductions reached its peak in the 2020–2022 period, when a multiplicative increase in RR = 1.03 (95% CI: 1.02–1.04, *p*-value < 0.001) substantially arrested the preceding decreasing trend. This indicates that there was no significant rise in stroke risk during the COVID-19 period. In terms of gender differentiation, males display a markedly elevated relative risk for hospitalisation owing to stroke (RR = 1.88, 95% CI: 1.86–1.90, *p*-value < 0.001) in comparison to females. No significant seasonal fluctuation has been detected following the adjustment for other variables, but there is a modest decrease in the summer season (RR = 0.98, 0.97–0.99, *p*-value = 0.003). The risk escalates significantly with age, particularly with respect to individuals aged 25–44 years, and there is an increased risk in individuals aged 45–65 (RR = 6.73, CI: 6.57–6.89, *p*-value < 0.001), aged 65–74 (RR = 28.82, CI: 28.19–29.46, *p*-value < 0.001), and those above 74 years (RR = 99.2, CI: 96.77–101.68, *p*-value < 0.001). With respect to the reference category 24–44 years, the risk of stroke hospitalisation is about 80% smaller for individuals aged 15–24 (RR = 0.21, CI: 0.20–0.22, *p*-value < 0.001), and about 90% smaller for individuals aged 0–14 (RR = 0.08, CI: 0.07–0.08, *p*-value < 0.001).

**Figure 3:**
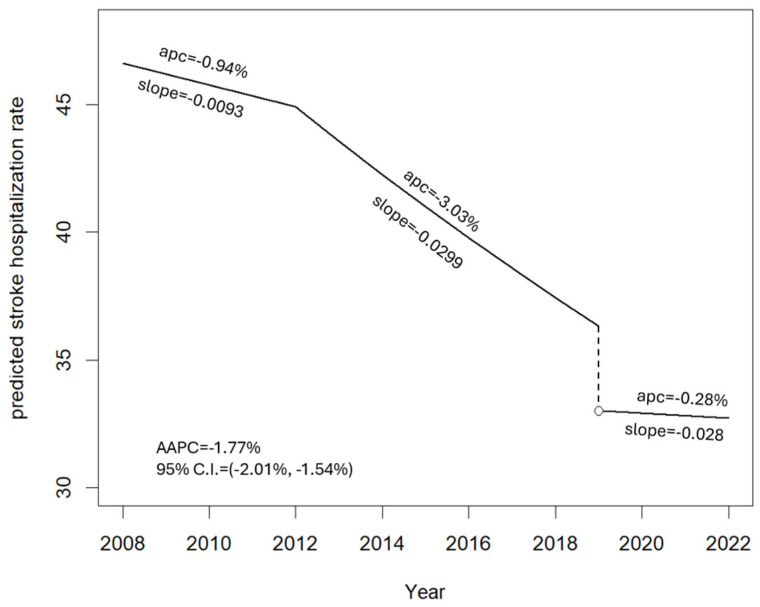
Predicted stroke hospitalisation rate with one change point (2012) and one jump and change point (2019) for an individual with male sex and age > 74 years in the spring season. The dashed line indicates the discontinuity of the regression in 2019.

Figure 3 illustrates the model forecasts of the stroke hospitalisation rate (×10,000 person years) over time, together with the APC and AAPC for an individual defined as follows: sex: male, age class: >74 years, and season: spring. The stroke hospitalisation rate experienced a notable annual decline of −1.77% (95% C.I. for the AAPC: [−2.01%, −1.54%]). However, the rate of decline (APC) fluctuated over time; a reduction of −0.94% per year was recorded from 2008 to 2012, which intensified to −3.03% per year from 2013 to 2019.

## 4. Discussion

The findings of this study provide important insights on stroke hospitalisation patterns and related risk factors in Italy from 2008 to 2022, covering about 1.7 million stroke-related hospitalisations. Our data appears to corroborate many conclusions that may assist policymakers in the efficient allocation of healthcare resources, including stroke units, rehabilitation services, and preventative initiatives, especially aimed at high-incidence regions or populations.

Specific risk variables that influence hospitalisation trends over time need significant attention. A notable sex-based disparity in stroke hospitalisation rates was identified, with males exhibiting a substantially elevated relative risk (RR = 1.87) for stroke hospitalisation relative to females, corroborating the observed sex-based difference in crude rates (19.96 per 10,000 person-years in males vs. 17.63 per 10,000 person-years in females). This trend aligns with prior studies, indicating that men typically exhibit higher stroke rates than women, presumably due to differences in cardiovascular risk profiles (e.g., increased prevalence of hypertension, smoking, and other risk factors among men). Numerous studies indicate that age appears to influence the impact of sex on stroke risk. Women are safeguarded against stroke until around 75–85 years of age, after which this protection diminishes compared to men [17].

The presence of risk factors, such as hypertension, diabetes, and atrial fibrillation, can differentially affect stroke risk in men and women. The higher frequency of hypertension and diabetes among hospitalised stroke patients can be attributed to the increased comorbidity rate in this population, as well as the higher incidence of large artery atherosclerotic strokes (LAAS), which are associated with moderate to severe neurological impairment [18].

The increased hospitalisation rate among stroke patients with atrial fibrillation is likely attributable to the higher prevalence of cardioembolic strokes in this group of patients. Patients with cardioembolic strokes exhibit greater disabilities and more severe immediate neurological impairment, likely associated with the higher magnitude of cardioembolic strokes compared to non-cardioembolic strokes [19].

This issue may elucidate the reported elevated hospitalisation rates in individuals with hypertension, diabetes, and atrial fibrillation.

Hypertension increases stroke risk by roughly 28% in males and 25% in women for each 10 mmHg rise in systolic blood pressure [20]. Furthermore, the risk of stroke for women doubles in the ten years subsequent to menopause. Changes in cardiovascular risk factors during menopause may exacerbate this increase and diminish the differences in risk between sexes as age progresses. Menopause is associated with elevated low-density lipoprotein (LDL) and triglyceride levels, reduced high-density lipoprotein (HDL) levels, changes in body fat distribution, fluctuations in insulin tolerance, heightened blood pressure, an increased incidence of metabolic syndrome, and elevated fibrinolytic and inflammatory markers [20]. The aforementioned risk factors may influence the relationship between hormonal fluctuations during the menopausal transition and stroke risk, as reduced levels of sex hormone-binding globulin and oestradiol, coupled with elevated levels of free androgen index, have been associated with risk factor levels during perimenopause [21].

A secondary topic of discussion is the significant rise in hospitalisation rates with advancing age. Individuals over the age of 74 have the greatest hospitalisation rates, significantly outpacing those of younger age groups. The risk increases significantly for individuals over 65, highlighting the crucial link between ageing and stroke risk, possibly influenced by factors such as cumulative vascular damage, comorbidities, and frailty. Studies indicate that the majority of strokes occur in individuals aged 65 and older, with an average age of onset of approximately 70 for males and 75 for females [22]. The increased risk is attributable to multiple factors, including an accumulation of chronic conditions, including hypertension, diabetes, atherosclerosis, atrial fibrillation, and hyperlipidaemia, which are more common and complex in older persons. Moreover, ageing is linked to psychological changes that may heighten vulnerability to cerebrovascular damage, including diminished vascular elasticity and increased arterial stiffness [23,24]. The outcomes of a stroke can differ markedly depending on the age of the patient. Older adults generally experience less favourable outcomes than younger individuals, with a higher risk of long-term disability and mortality [20]. This is partially due to the presence of comorbidities and the reduced recuperative capacity of the brain in advanced age [25].

After adjusting for confounding factors such as sex and age, our data indicates an overall decline in hospitalisation rates over time, with a reduction of approximately 20% over 15 years. The Italian Ministry of Health corroborates this trend, attributing the decrease in the incidence and mortality of cerebrovascular events, including strokes and transient ischaemic attacks (TIA), to multiple factors [26].

This trend may indicate several reasons, including improvements in stroke prevention and management strategies, improved control of risk factors such as hypertension, advancements in acute stroke care, changes in lifestyle, and a gradual decrease in hospital beds in Italy [27]. This latter has specifically responded to multiple pressures, including economic austerity, healthcare reform, and the demand for more efficient and specialised care. Nonetheless, it must be acknowledged that the relationship between bed reduction and stroke-related hospitalisation may be intricate and affected by various other factors that illustrate the interaction of these trends. Italy implemented numerous public health programmes to promote better lifestyles, reduce smoking, manage hypertension, and advocate for exercise and proper diets [28]. These initiatives have reduced the incidence of risk factors, like hypertension, diabetes, and hyperlipidaemia, which are significant contributors to stroke. The substantial usage of antihypertensive drugs, statins, and anticoagulants may significantly contribute to the prevention of strokes [29]. The implementation of more efficient hospital care pathways and emergency care may have diminished the necessity for hospitalisation for a number of patients [30].

Additional consideration should be provided to the significant reduction in stroke-related hospitalisations during the COVID-19 pandemic. In this sense, certain hospital admissions for non-COVID conditions, including stroke, might have been reduced, due to disruptions in the healthcare system or the reluctance of patients to pursue medical care during the pandemic [31]. Multiple research studies support this reduction in hospitalisations, by demonstrating that the pandemic hindered the treatment of illnesses not associated with COVID-19 [13,32,33]. Moreover, a study conducted in Romania revealed that the COVID-19 pandemic caused longer treatment times for patients with ischaemic stroke presenting to emergency rooms; the door-to-needle time increased from 43 min in 2020 to 60 min in 2023 [34]. It is essential to point out that our study revealed no statistically significant association between COVID-19 and a higher risk of hospitalisation for stroke, despite the assertion that stroke may be a serious consequence of the virus. This absence of association may be attributed to the fact that COVID-19 pandemic resulted in a notable decline in hospital admissions, potentially due to individuals delaying seeking medical attention for ischaemic stroke, either from fear of virus exposure or owing to overwhelmed healthcare facilities. These factors may have influenced the lack of a direct correlation between COVID-19 and increased stroke-related hospitalisations in our study.

A major limitation of the study is the potential misclassification of stroke, resulting from the erroneous or inconsistent use of ICD-9-CM codes, which could negatively impact the accuracy of stroke identification. Nonetheless, some authors have documented a comparatively high accuracy of these ICD-9-CM codes in diagnosing approximately 90% of individuals with this medical condition [35].

Furthermore, the findings are derived from an Italian cohort and may not be fully generalisable to populations in countries with different healthcare systems or sociodemographic profiles.

Although regional variability was included as a random effect, the study does not examine specific regional factors, such as healthcare policy differences, that may influence stroke hospitalisation rates.

Lastly, the assessment of factors potentially contributing to strokes may be incomplete, and the study is limited by the inability to account for additional important confounding factors, especially due to the lack of detailed clinical information which might bias the results.

## 5. Conclusions

In conclusion, the study highlights key factors that may influence stroke hospitalisations, notably the roles of age, sex, time, and the potential impact of the COVID-19 pandemic. The significant age-related increase in risk underscores the importance of targeted interventions for older adults. While the observed decline in overall rates may reflect improvements in stroke prevention and management, alternative explanations cannot be excluded. Furthermore, the findings suggest that healthcare disruptions during the pandemic could have influenced stroke hospitalisation patterns, a phenomenon that requires further investigation to draw definitive conclusions.

## Figures and Tables

**Table 1:** Characteristics of hospitalisation due to stroke in Italy 2008–2022.

Variable		Hospitalisation Cases	Person-Year	Rates × 10,000 Person Years
All		1,689,844	900,752,318	18.76
Season	Autumn	422,714	225,188,080	18.77
	Summer	407,842	225,188,080	18.11
	Winter	430,043	225,188,080	19.10
	Spring	429,245	225,188,080	19.06
Sex	F	816,542	463,150,979	17.63
	M	873,302	437,601,339	19.96
Age Class	0–14	1187	122,562,120	0.10
	15–24	2203	89,050,229	0.25
	25–44	28.69	237,121,101	1.21
	45–64	244,063	256,147,143	9.53
	65–74	370,435	97,419,241	38.02
	>74	1,043,266	98,452,484	105.97
Hospitalisation year	2008	118,689	59,619,290	19.91
	2009	119,279	60,045,068	19.86
	2010	123,344	60,340,328	20.44
	2011	123.19	60,626,442	20.32
	2012	121,478	59,394,207	20.45
	2013	121,032	59,685,227	20.28
	2014	119,449	60,782,668	19.65
	2015	117,797	60,795,612	19.38
	2016	116.89	60,665,551	19.27
	2017	109,594	60,589,445	18.09
	2018	109,742	60,483,973	18.14
	2019	106,634	59,816,673	17.83
	2020	93,533	59,641,488	15.68
	2021	94,052	59,236,213	15.88
	2022	95,141	59,030,133	16.12

**Table 2:** Model estimates of the multivariable PIG model. The reference category is sex = F, season = spring, age class = 25–44 years.

	Estimate	Std. Error	*t* Value	Pr (>|*t*|)
(Intercept)	−10.509	0.012	−843.229	<0.001
Slope for 2008–2012	−0.009	0.002	−5.119	<0.001
I (year > 2019)	−0.096	0.013	−7.558	<0.001
Slope difference for 2008–2012	−0.021	0.003	−8.146	<0.001
Slope difference for 2008–2012	0.028	0.006	4.942	<0.001
Sex = M	0.629	0.006	112.607	<0.001
Season = summer	−0.016	0.005	−2.985	0.003
Season = winter	−0.002	0.005	−0.343	0.732
Season = spring	0.010	0.005	1.935	0.053
Age class 0–14	−2.553	0.040	−63.205	<0.001
Age class 15–24	−1.567	0.033	−47.949	<0.001
Age class 45–64	1.906	0.012	159.190	<0.001
Age class 65–74	3.361	0.011	297.627	<0.001
Age class > 74	4.597	0.013	364.031	<0.001

## Data Availability

The raw data supporting the conclusions of this article will be made available by the authors on request.

## Supplementary Materials

The following supporting information can be downloaded at: https://www.mdpi.com/article/10.3390/jcm14020353/s1, Statistical details about the model parameter interpretation and the calculation of the ACP and AACP and their confidence intervals.
