# Supplementary material for: Evolution of Hospitalisation Due to Stroke in Italy Before and After the Outbreak of the COVID-19 Epidemic: A Population-Based Study Using Administrative Data
Source: J Clin Med. 2025 Jan 8;14(2):353. doi: 10.3390/jcm14020353 (PMC11765534; doi:10.3390/jcm14020353)
Supplement: Supplementary file 1 [file jcm-14-00353-s001.zip › jcm-3356893-supplementary.pdf]

Journal of Clinical Medicine

Supplementary Materials

Evolution of Hospitalisation Due to Stroke in Italy Before and After the Outbreak of the COVID-19 Epidemic: A Population-Based Study Using Administrative Data

Emanuele Amodio [1], Gabriele Di Maria [1], Manuela Lodico [1], Dario Genovese [1], Vito M. R. Muggeo [2], Laura Maniscalco [1], Michela Conti [3], Maria Sergio [1], Antonio Cascio [1], Antonino Tuttolomondo [1], Domenica Matranga [1], Francesco Vitale [1] and Marco Enea [1,*]

1 Department of Health Promotion, Mother and Child Care, Internal Medicine and Medical Specialties, University of Palermo, 90127 Palermo, Italy; emanuele.amodio@unipa.it (E.A.); gabriele.dimaria@unipa.it (G.D.M.); manuela.lodico@you.unipa.it (M.L.); dario.genovese@unipa.it (D.G.); laura.maniscalco04@unipa.it (L.M.); maria.sergio@unipa.it (M.S.); antonio.cascio03@unipa.it (A.C.); antonino.tuttolomondo@unipa.it (A.T.); domenica.matranga@unipa.it (D.M.); francesco.vitale@unipa.it (F.V.)
2 Department of Economics, Business and Statistics, University of Palermo, 90128 Palermo, Italy; vito.muggeo@unipa.it
3 Azienda Ospedaliera Ospedali Riuniti (AOOR) Villa Sofia Cervello, 90146 Palermo, Italy; michelaconti@virgilio.it
* Correspondence: marco.enea@unipa.it

Model Specification

The linear predictor for the location parameter of the gamlss Poisson Inverse Gaussian (PIG) model is the following:

$$\log\left(\frac{\mu_{ij}}{pop}\right) = \beta_0 + year_i \times \beta_1 + (year_i - 2012)_+ \times \beta_2 + I(year_i > 2019) \times \beta_3 + (year_i - 2019)_+ \times \beta_4 + sex_i \times \beta_5 + summer_i \times \beta_6 + winter_i \times \beta_7 + spring_i \times \beta_8 + ageclass_{i,0-14} \times \beta_9 + ageclass_{i,15-24} \times \beta_{10} + ageclass_{i,45-64} \times \beta_{11} + ageclass_{i,65-74} \times \beta_{12} + ageclass_{i,>74} \times \beta_{13} + \gamma_{ij}$$

where:

- i and j are the subject (record) and region indices, respectively;
- β_1 is the slope for the years 2008–2012;
- $\beta_1 + \beta_2$ is the slope for the years 2013–2019, being β_2 the difference in slope for the years 2013-2019 with respect to the years 2008–2012, and $(year_i - 2012)_+$ being the change point of the slope for the years 2013–2019;
- β_3 quantifies the "jump" between the years 2019 and 2020;
- $\beta_1 + \beta_2 + \beta_4$ is the slope for the years 2020–2022, being β_4 the difference in slope for the years 2020-2022 with respect to the years 2013–2019.
- γ_{ij} is the random effect of the region.

The baseline variable values are year = 2008, sex = F, season=autumn, and ageclass = 25-44.

The Annual Percentage Change (APC) from the model, reported in Figure 3, is calculated as follows:

$$APC_{2008-2012} = [\exp(\beta_1) - 1] \times 100,$$

 https://doi.org/10.3390/xxxxx

$$APC_{2013-2019} = [\exp(\beta_1 + \beta_2) - 1] \times 100,$$

$$APC_{2020-2023} = [\exp(\beta_1 + \beta_2 + \beta_3) - 1] \times 100,$$

while the Average Annual Percentage Change (AAPC) is calculated as:

$$w_{2008-2012} \times \beta_1 + w_{2013-2020} \times (\beta_1 + \beta_2) + w_{2020-2022} \times (\beta_1 + \beta_2 + \beta_4),$$

were

$$w_{2008-2012} = \frac{5}{15}, w_{2013-2019} = \frac{7}{15}, w_{2020-2022} = \frac{3}{15}.$$

The standard error calculation of the AAPC is based on:

1) the vector $w = (w_{2008-2012}, w_{2013-2020}, w_{2020-2022})'$ of weights;
2) the 3×3 sub-matrix B of the model variances/covariances matrix V, containing the variances/covariances for β_1, β_2, and β_4 respectively;
3) a contrast matrix M such that $M = \begin{bmatrix} 1 & 1 & 1 \\ 0 & 1 & 1 \\ 0 & 0 & 1 \end{bmatrix}$.

Then, the AAPC's standard error is calculated as $se = (w'MBM'w)^{\frac{1}{2}}$. Finally, the 95% confidence interval for AAPC is calculated as $AAPC \pm 1.96 \times se$.
